# Follow-Up of Neonatal Hearing Screening in the Risk Factor Group for Hearing Loss: Results from a Tertiary Medical Center

**DOI:** 10.3390/children11111336

**Published:** 2024-10-31

**Authors:** Miriam Geal Dor, Menachem Gross, Cahtia Adelman

**Affiliations:** 1 Speech & Hearing Center, Hadassah Hebrew-University Medical Center, Jerusalem 91120, Israel; cahtiaa@hadassah.org.il; 2 Department of Communication Disorders, Hadassah Academic College, Jerusalem 91010, Israel; 3 Department of Otolaryngology Head and Neck Surgery, Hadassah Hebrew-University Medical Center, Jerusalem 91120, Israel; gross@hadassah.org.il; 4 Faculty of Medicine, Hebrew University, Jerusalem 91120, Israel

**Keywords:** neonatal hearing screening, follow up, risk factor, monitoring audiologic tests, hearing loss

## Abstract

Introduction: Universal newborn hearing screening has been successfully implemented in many places around the world, and it is recommended that cases with risk factors for hearing loss be followed-up regardless of hearing screening results. However, there is a need for clarity regarding the recommended rate of follow-up and which tests should be performed. The aim of this study was to assess the audiologic follow-up program for the group with risk factors. Method: Our retrospective study involved children of various ages with a risk factor for hearing loss who passed the initial neonatal hearing test but were later diagnosed with hearing loss. Out of 113,708 children born at Hadassah University Medical Center during the years 2013–2021, 6763 were at risk of hearing loss, and their follow-up audiologic test results were studied. Results: Audiologic testing including ABR, OAE, tympanometry and behavioral audiometry was performed in 1534 of 6763 (23%) of the risk factor group that returned to the hospital. In total, 73 children (4.7%) were diagnosed with hearing loss, 54 of whom failed the initial screening and 19 who had passed it. Further examination of the children that passed the initial screening and were later diagnosed with a hearing loss revealed that four cases had been missed in screening (one familial mild hearing loss, one familial progressive loss, one premature infant with a high tone loss, and one NICU graduate with CNS involvement). Another nine cases had late-onset hearing loss (three meningitis, five CMV, and one with a mitochondrial disease). An additional six cases were diagnosed late, and the age of onset of the hearing loss was unknown (two intubated, two with hydrocephalus, one with Cerebral Palsy, and one with general developmental delay). Conclusions: These results reveal the importance of implementing a refined protocol for monitoring hearing in the high-risk group of children that pass neonatal hearing screening with respect to which hearing tests should be conducted, at what age, and the duration of follow-up. Also, barriers to follow-up must be dealt with, and parents should be more involved in the monitoring process.

## 1. Introduction

Early diagnosis of and interventions for hearing loss are important to ensure adequate language development, communication, social and emotional skills [1]. Therefore, neonatal hearing screening programs have been established worldwide to identify hearing loss as early as possible. In 2019, the Joint Committee for Infant Hearing (JCIH) presented the “1-3-6” guidelines: achieve screening by 1 month, diagnosis by 3 months, and amplification as well as enrollment into early intervention by 6 months, while further encouraging to strive to reach a “1-2-3” timeline, highlighting the importance of timely diagnosis [2].

Neonates who do not pass the initial screening are usually referred for to audiology clinics for diagnosis. Furthermore, it is recommended that neonates with risk factors for hearing loss should be followed up in audiology and otolaryngology clinics, regardless of their hearing screening results. Follow-up is essential for the prevention of missed diagnoses in a population that has a high prevalence of hearing loss. Despite the importance of early intervention, follow-up in the group of high-risk infants remains a significant issue with respect to the monitoring schedule, as it is still unclear how often should they be tested and which hearing tests should be performed. Moreover, the at-risk population is a heterogeneous group. Not all risk factors are considered equally risky, and some of the factors are associated with greater concern for progressive and delayed onset hearing loss, and therefore should be monitored more persistently [3].

The JCIH [2] presented new guidelines for infants who pass the hearing screening and require further diagnostic audiologic testing due to several risk factors. In cases of CMV (cytomegalovirus), Zika and ECMO (Extracorporeal Membrane Oxygenation), Auditory nerve- and Brainstem-evoked Response (ABR) testing is recommended by 3 months of age and monitoring should take place once a year or at shorter intervals based on caregiver concern. However, in cases of familial hearing loss, a NICU (Neonatal Intensive Care Unit) stay longer than 5 days, hyperbilirubinemia with exchange transfusion, aminoglycoside administration for over 5 days, in utero infection (i.e., syphilis, herpes, toxoplasmosis), craniofacial malformation, microcephaly or hydrocephalus, and syndromes associated with hearing loss, the recommendation is to undergo hearing evaluation by the age of 9 months.

The Public Health guidelines in England [4] suggest targeted surveillance and audiological monitoring for the population with risk factors that passed newborn hearing screening. A recommendation for ABR testing was made in cases with microtia or atresia, meningitis, and CMV within 4 weeks or sooner even if the infants passed the hearing screening. Behavioral testing at around 8 months or sooner is recommended in cases of syndromes associated with hearing loss, craniofacial abnormalities, and congenital infection such as toxoplasmosis or rubella. The choice of testing at 8 months was made on the pragmatic basis that at this age, most infants are developmentally ready for a behavioral assessment of hearing which is typically a straightforward procedure that can be accomplished. After a major review of new evidence, some categories were removed from the list for targeted follow-up, such as familial hearing loss, hyperbilirubinemia, mechanical ventilation, and neurodevelopmental disorders [5].

Traditionally, targeted surveillance has been proposed as the primary method for monitoring hearing in children who may be at risk for developing hearing loss. Although the principles remain the same, because of the excessive burden on both families and audiology services, significant changes have been made to the risk factors included in the list and in the frequency of hearing assessments, according to the results of further re-search [6]. Although it is strongly recommended that children with hearing loss risk fac-tors return for follow-up regardless of the hearing screening outcome, it is critical to determine the results of follow-up for children with risk factors in order to make appropriate recommendations for continued follow-up procedures [7].

The primary aim of this study was to assess the follow-up program of the group at risk for hearing loss to assess how many children with risk factors were diagnosed with a hearing loss, at what age, and to which category of risk factor they belonged. The secondary aim was to determine how many returned for follow-up regardless of hearing screening results, in order to re-evaluate the effectiveness of the follow-up protocol for identification of hearing loss.

## 2. Methods

This study was retrospective, utilizing evaluation of the medical files of infants born at a tertiary medical center between 2013 and 2021. Of 113,708 newborns, 6763 had risk factors for hearing loss as defined by the JCIH 2007 list [8]. Then, the files of all 6763 infants were reviewed to see if they had returned for follow-up at our speech and hearing clinic. The following data were documented for the 1534 subjects that returned:

1. Risk factors using the classification of the JCIH [8]: NICU stay >5 days including hyperbilirubinemia, aminoglycosides, hypoxia and ECMO; familial hearing loss, int utero infections such as CMV; skin tag; craniofacial malformation; syndromes associated with hearing loss; and infections associated with hearing loss such as meningitis.
2. Results of hearing screening.
3. Length of follow-up: age of first and last follow-up.
4. Hearing tests performed–ABR, OtoAcoustic Emissions (OAEs), behavioral testing, tympanometry, etc.
5. Hearing status—normal hearing, (up to 15 dB in 500–4000 Hz), age-appropriate hearing, conductive hearing loss, sensorineural hearing loss, and a group in which the results were inconclusive.

The study was approved by the Institutional Review Board (HMO 0244-21). Descriptive statistics were used to analyze the data.

## 3. Results

During the years 2013–2021, the risk factor group included 6763 infants, of which 6329 (93.6%) had passed the initial hearing screening before discharge from the hospital, while 434 (6.4%) had failed the initial screening. Since this was the high-risk group, regardless of screening results, all received recommendations to continue follow-up.

**Figure 1:**
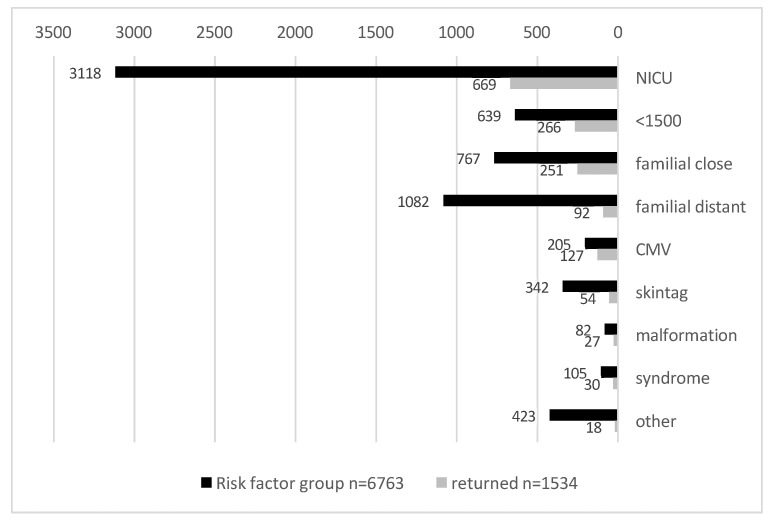
The distribution of the different risk factor categories with the rate of infants that returned for follow-up in each risk factor group.

*Risk factors:* The distribution of the risk factors in 6763 infants is displayed in Figure 1. The most common risk factor was for graduates of neonatal intensive care, with 46% having stayed in the NICU for over 5 days, and an additional 9% were not only NICU graduates but were also born at a very low birth weight (LBW) of <1500 g. Another large group of 11% had a history of familial hearing loss in their close family (parents and siblings) as a risk factor, while another 16% had a history of familial hearing loss in the extended family. Less common risk factors included CMV in 3% of the infants, skintags in 5%, craniofacial malformations in 1%, and syndromes associated with hearing loss in 2% while other risk factors were present for 6%. In cases in which there was more than one risk factor, they were assigned to the more specific category (for example, a newborn with a history of CMV and a NICU stay will appear in the CMV group).

Figure 1 shows those of the 6763 infants that returned for follow-up in each of the risk factor groups. Only 23% (1534) returned for follow-up in our clinic. With respect to the specific risk groups, the rate of return for follow-up was higher for patients with the following risk factors: CMV (62%), LBW (42%), malformation (33%), close familial hearing loss (33%), syndromes (29%), and a previous NICU stay (21%), than for other high-risk groups: skin tag (16%), distant familial hearing loss (9%), and others (4%).

**Figure 2:**
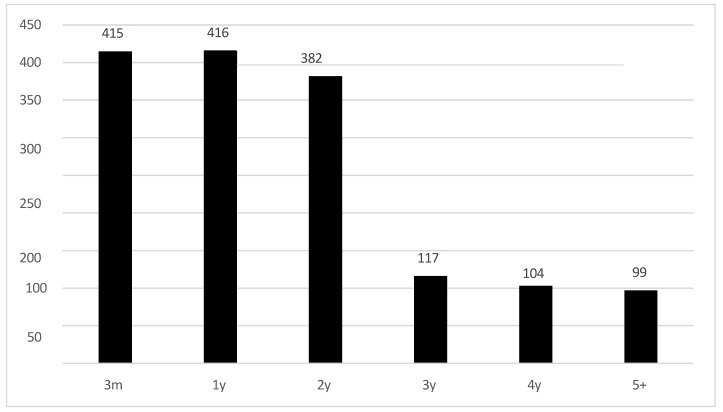
Length of follow-up by age of the 1534 subjects that returned in the risk factor group (m = months; y = years).

The duration of follow-up by age of the subjects in the risk factor group is demonstrated in Figure 2. Over half of the children (54%) returned for follow-up during the first year of life, another 25% continued follow-up until the age of 2 years, 8% continued follow-up until age 3, and only 13% continued for longer.

*Neonatal Hearing Screening:* In the group that returned for follow-up (*n* = 1534), the number of children that passed the neonatal hearing screening was 1230 (80%) while the number of infants that failed was 304 (20%).

**Figure 3:**
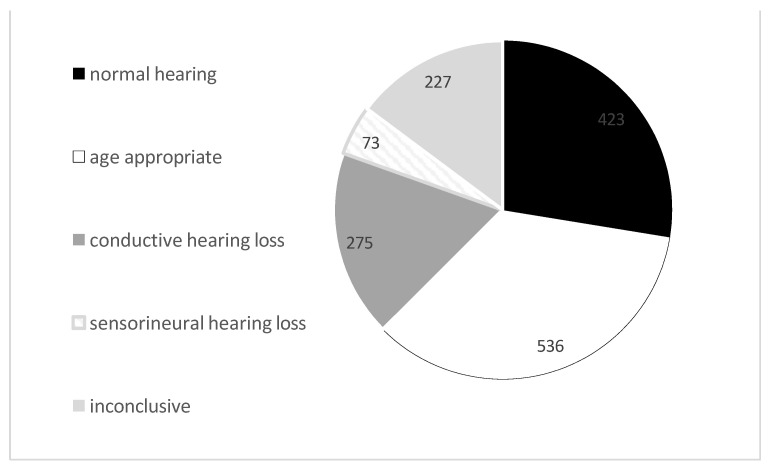
Hearing evaluation results of the 1534 subjects that returned for follow-up in the risk factor group.

*Hearing evaluation results:* Figure 3 shows the hearing evaluation results of 1534 sub-jects who returned for follow-up in the risk factor group. A total of 27% were found to have hearing within normal limits, 35% had appropriate-for-age hearing results (for example, a child that returned for behavioral testing at the age of 2 years, whose speech reception threshold (SRT) was within normal limits, but who did not cooperate for a complete audiogram including all frequencies), 18% had non-permanent conductive hearing loss, in 15% the hearing test results were inconclusive (for example, a 9-month-old infant that came for a behavioral test. In the free field, his voice detection level was 30 dB, which can indicate mild hearing loss, and his tympanometry was B, which is in line with the ENT observation of SOM. However, as can occur at this age, he did not cooperate with testing through phones or a bone vibrator, nor did he respond to tonal stimuli, so it is unclear whether his was only a conductive hearing loss) and further follow-up was recommended.

Seventy-three children (4.7%) were found to have sensorineural hearing loss and were referred for rehabilitation (hearing aids, cochlear implant, and speech therapy). The percentages were pretty similar in the group that failed or passed the initial screening: most (30% and 29%, respectively) were found to have normal hearing, age-appropriate hearing (18% and 34%, respectively), non-permanent conductive hearing loss (15% and 17%, respectively) or inconclusive audiologic results (19% and 14%, respectively).

In the group with sensorineural hearing loss, 54 infants (74%) failed the initial neonatal hearing screening, but 19 infants (26%) passed the neonatal hearing screening protocol which included both transient otoacoustic emission and automated ABR (AABR). The average age of patients diagnosed with sensorineural hearing loss was just over 4 months for the infants that failed the screening. In contrast, the average age of diagnosis of hearing loss for the group that passed the screening OAE + AABR was much later, at the age of 16 months.

Table 1 displays in-depth information about the children diagnosed with a sensorineural hearing loss. As can be seen in the group that passed the screening, the distribution of the risk factor was as follows: two had a family history of hearing loss (one mild case of hearing loss and one progressive case); of the four NICU graduates, one premature infant had a high tone loss that can be missed during hearing screening, and one with Cerebral Palsy, one with CNS involvement, and one with a mitochondrial disease had late-onset hearing loss. Of the five cases with very low birth weight, two cases had a history of asphyxia, two had a history of hydrocephalus, and one was diagnosed with a general developmental delay, and of the additional cases diagnosed with late-onset hearing loss, three were a result of meningitis and five were caused by CMV.

## 4. Discussion

It is well known that early identification of hearing loss is important for early intervention strategies. Delays in confirmation and rehabilitation in cases of hearing loss are of concern because of the potentially negative effects on the development of language and communication, as well as emotional, social, and academic skills [1].

It is widely acknowledged that children with risk factors should be closely monitored for hearing loss regardless of their results on neonatal hearing screening. Previous studies [7,9,10] demonstrated that although screening protocols with OAE and AABR had a good sensitivity in predicting hearing loss, the sensitivity was somewhat lower in the group of NICU graduates. Therefore, the researchers aimed to provide recommendations about who needs full diagnostic hearing testing based on the best available evidence [9].

### 4.1. Responsiveness and Duration of Follow-Up

One key concern in our study is that most infants did not continue after initial screening and were lost to follow-up. Dumanch et al. (2017) [7], reports that although many resources are dedicated to alerting parents of children with risk factors to return for follow-up, in several programs, only one-third of the children with risk factors have a documented follow-up. In another study [5], the authors found that just over half (55%) the children attended a follow-up appointment. In a program described by McInerney et al. (2020) [3], compliance with the first follow-up appointment was very high (85%). Although these results appear impressive at first glance, only 10.3% continued to return for follow-up appointments and completed the study protocol.

In our study, only 23% of patients returned for follow-up at our center, and most returned during the first year of life only. It is very possible that some have continued follow-up elsewhere in the community rather than at our hospital clinic. If so, the rates and length of follow-up may in reality be higher than documented here, and therefore may have been underestimated in our study. However, it may be that follow-up monitoring of hearing is not considered important by the families of the children. As the follow-up rate of children with risk factors who passed the newborn hearing screening is much lower than that of those who did not pass initial screening, it is critical to examine factors which can be related to loss to follow-up in order to make appropriate recommendations for continuing care [7].

Cheung et al. (2022) [11] suggest that the socioeconomic and demographic factors of the parents may have an influence on the “lost to follow-up” population. Another barrier associated with those lost to follow-up may be lack of awareness of the importance of follow-up testing [3]. Others [5] suggest this may be related to the reassurance families felt based on the results of the newborn hearing screening, coupled with their own observations of their child’s response to auditory stimuli. Also, some of these children presumably have other medical conditions necessitating more frequent appointments, and it may be that hearing is not perceived as a priority.

In a study conducted by Glick et al. (2017) [12], poor follow-up attendance was directly related to parents’ understanding and knowledge of the role of hearing in development, especially in children with multiple diagnoses. This demonstrates the need to provide better education and counseling for families and other health care providers regarding the association between risk factors and hearing loss and the consequences of “loss to follow-up”. It is important to further study and take into consideration how the results of hearing screening are presented and how they are comprehended by parents in order to improve follow-up attendance [3].

### 4.2. Classification of Risk Factors

The importance of developing protocols for hearing loss audiologic assessment after the stage of newborn screening must not be underestimated. Although the JCIH [2] has traditionally recommended monitoring risk factors in order to detect postnatal hearing loss by 3 months (in cases of CMV, meningitis, and ECMO) or by 9 months (in cases of familial hearing loss, NICU stay, malformations, and syndromes), and continued monitoring, the implementation of a long term follow-up program is still a decision that must be made by each center. Despite limited resources, there is a need for further investigation of the influence of the various risk factors on late-onset hearing loss in order to ensure timely audiological evaluation and early intervention [11]. McInerney et al. (2020) [3] state that a conservative approach to monitoring is often pursued in order to ensure that infants who develop hearing loss are identified early. However, this may lead to over-burdening of audiology facilities and of families. Therefore, these researchers conclude that it may be more sensible to focus long-term continued monitoring efforts on a smaller group of infants by using an approach of classifying risks into specific subgroups that necessitate more intense and earlier follow-up, or less intense later follow-up.

The results of our study demonstrate the need to establish a follow-up protocol that is dependent on specific risk factors. While cases with a high probability of progressive hearing loss (such as meningitis or CMV) should be followed up closely, we suggest that cases with lower probability (i.e., distant familial hearing loss, skintags) may need less frequent follow-up appointments.

In previous studies, the risk factors most frequently associated with later-identified hearing loss after passing initial hearing screening were CMV [5,6,13], NICU stay [5,14,15], respiratory episodes such as mechanical ventilation, asphyxia and ECMO support [6,14,16,17], craniofacial anomalies [5,13,14], syndromes [5,18], and neonatal encephalopathy [17]. Other risk factors were not associated with late-onset hearing loss and may not need further long-term monitoring. These include skintags, low birth weight, toxoplasmosis [6], hyperbilirubinemia, congenital herpes, syphilis, and rubella [3]. There is disagreement with respect to familial hearing loss; while some [18] found a high rate of children who passed screening were later diagnosed with hearing loss, others [5] found that passing screening was not associated with late-onset hearing loss.

The different studies show there is still no general agreement as to the list of risk factors which require closer monitoring, and there is a need for further investigation to gather the evidence necessary for the creation of a continuous long-term hearing follow-up protocol based on monitoring according to specific factors, with a timeline of how long patients should be followed.

### 4.3. Hearing Loss and Age of Diagnosis

In our study, 62% of participants had normal or behavioral age-appropriate hearing test results, an additional 18% had non-permanent conductive hearing loss, and 4.7% had a sensorineural hearing loss.

In a similar study [7], it was shown that 96% of all infants with risk factors had been found to have hearing within normal limits by the age of 3 years, possibly showing in retrospect that many evaluations had been unnecessary. However, for the 4% diagnosed with hearing loss, the follow-up was invaluable, and therefore may justify the monitoring program.

In a follow-up design described by Wood et al. (2013) [5], 77% of children had a recorded outcome of satisfactory hearing in both ears at their last assessment and 0.35% had permanent hearing loss; the remaining children had a conductive hearing impairment or a mild hearing loss that did not meet the criterion for the proposed screening target condition.

Discovering the 4.7% of children with sensorineural hearing loss was the main goal of the monitoring. However, most of these 4.7% had already failed the newborn hearing screening, as only 26% of the group with hearing loss passed the initial hearing screening and were diagnosed only during follow-up appointments. There is a need to further ascertain the specific risk factors that were diagnosed only during the follow-up program in order to refine the recommendations for monitoring.

The poor follow-up rates, as mentioned earlier, likely contribute to an underestimation of true prevalence [14]. Thus, the true incidence of hearing loss in this population may be higher and underrepresented in the studies reported.

Another subgroup in our study was the 15% of children in whom the hearing test results were inconclusive, mostly due to lack of cooperation with a complete behavioral hearing test at a younger age. The issue of inconclusive hearing tests should be further addressed so that hearing loss diagnosis is not delayed.

When comparing the age of diagnosis of the subgroup that failed and the subgroup that passed initial hearing screening, it was noticed in the present study that while the group that initially failed hearing screening was diagnosed within the JCIH [2] recommendation time window of “1-3-6”, the second group that initially passed the hearing screening was diagnosed at a later age, and therefore began rehabilitation beyond the recommended timeline. This was similar to the findings of previous reports [18,19].

These studies suggest possible reasons for late diagnosis of hearing loss, despite the early initiation of the assessment process: the presence of multiple developmental issues or complex medical issues, children who also had middle-ear dysfunction at the time of assessment, complicated family follow-up concerns, and mild hearing loss. In our group of 19 who had passed the initial screening, 2 were diagnosed with a mild hearing loss, and an additional child was diagnosed with a high tone loss, and as is well known [20], these cases do not meet the criterion for the screening target, and therefore are missed. An additional nine cases had a documented late onset, and therefore could not have been diagnosed earlier. However, an additional seven children had passed the initial screening and were later diagnosed with hearing loss that might have been present for some time and nevertheless was missed. It is impossible to retrospectively recognize the exact age of hearing loss onset, since the age of identification can very well be delayed. Wilding et al. 2024 [18] raise the question as to whether there is any benefit of the risk factor follow-up, since the median age of hearing loss confirmation in the group of high risk that passed initial screening was similar to those in the general population with late-onset hearing loss without any referral.

### 4.4. Study Limitations

The major concern in our study is the missing data in the “lost to follow-up” group. It is important to mention that all parents received oral and written recommendations for follow-up when their children were discharged from the hospital. It may very well be that many continued follow-up elsewhere at communal clinics rather than at the hospital. The main focus of this study was not the lost to follow-up population; however, it is important to design a continuous study to investigate and learn about the challenges that exist in order to avoid or decrease the numbers of children “lost to follow-up”. For example, it would be of interest to find out if parents of NICU babies do not follow-up because of the complexity of the medical situation or because they did not comprehend the information about risk factors for hearing loss due to the manner and time of presentation and explanation of the information.

Another concern may be a bias if risk factors are present but not recorded accurately, and therefore, after the initial pass, the children are not referred for follow-up at a later date. It is hard to know if and how many such cases exist. This would mainly occur if parents did not report familial hearing loss, and these cases cannot be determined.

The third concern is the group of children with inconclusive hearing test results. As is well known, cooperation of young children in behavioral audiometry hearing tests is incomplete and therefore threshold determination may be uncertain. In many infants, the results obtained did not include ear- or frequency-specific information. Behavioral testing using visual reinforcement audiometry (VRA) is not always straightforward, and protocols for VRA are not well standardized [21]. Therefore, it is often impossible to conclude whether hearing is within normal limits. The limitation of cooperation in behavioral testing is well known in the pediatric clinic. The choice of whether to continue with behavioral audiometry or ABR with sedation should be based on the clinician’s impression of the possibility of cooperation upon further testing.

Wilding et al. (2024) [18] state that considerable resources are required for follow-up appointments, together with parental counseling. However, the late age of identification in this group raises the question of reconsidering the protocol for early identification of hearing loss. Lu et al. (2011) [22] state that the rate of delayed-onset hearing loss is 0.75/1000 in a population of 3–6-year-old children who had previously passed the new-born hearing screening and may not have any risk factors. Therefore, they suggest that hearing assessment should not be restricted to children with risk factors; rather, it would be better to extend hearing screening to the entire preschool-age population. Beswick et al. (2012) [6] state that second-phase screening programs may be an additional or alternative strategy for targeted risk factor monitoring in the future, but they require evidence-based, large-scale, population-based research as well as investigation of the cost–benefit of the program. A later-stage (at 7–9 months old) behavioral hearing screening was performed in the past in our country [23] and discontinued with the advent of national neonatal screening about 15 years ago. Perhaps adding a second stage screening for the entire population should be reconsidered.

Meanwhile, in addition to refining the risk-factor protocol, it is important to emphasize to parents and professionals the importance of monitoring developmental milestones and auditory skills, as well as remaining alert to the possibility of later-onset hearing loss, and ensuring prompt referral for audiological assessment in the event of any concern [2,5,6].

In summary, universal newborn hearing screening programs have been successfully implemented [24]. This success has not yet been achieved in programs monitoring late-onset hearing loss. There is a need to continue to explore ways to implement these same strategies efficiently.

We conclude that it is important to better define a specific and refined protocol for monitoring of hearing in the risk-factor group children that pass neonatal hearing screening, with respect to which hearing tests should be conducted, at which age, and the overall duration of follow-up. However, the high percentage of children lost to follow-up must be reduced. There is a further need to address the barriers to follow-up and integrate an approach that engages parents in the monitoring process.

## Figures and Tables

**Table 1:** Risk factor group of infants diagnosed with sensorineural hearing loss.

Failed Initial Hearing Screening *n* = 54		Passed Initial Hearing Screening *n* = 19
NICU	11	4
<1500 g	4	5
Familial HL (close relation)	22	2
Familial HL (distant relation)	2	
CMV	6	5
Malformation	1	
Syndrome	7	
Meningitis	1	3
Age of confirmation of diagnosis (months)	4.2 ± 2.4	16.4 ± 11.2

## Data Availability

Data are contained within the article.
